# Subterahertz dielectric relaxation in lead-free Ba(Zr,Ti)O_3_ relaxor ferroelectrics

**DOI:** 10.1038/ncomms11014

**Published:** 2016-04-04

**Authors:** D. Wang, A. A. Bokov, Z.-G. Ye, J. Hlinka, L. Bellaiche

**Affiliations:** 1 Electronic Materials Research Laboratory—Key Laboratory of the Ministry of Education and International Center for Dielectric Research, School of Electronic and Information Engineering, Xi'an Jiaotong University, No. 28, Xianning West Road, Xi'an 710049, China; 2 Department of Chemistry and 4D LABS, Simon Fraser University, Burnaby, British Columbia, Canada V5A 1A6; 3 Department of Dielectrics, The Czech Academy of Sciences, Na Slovance 2, CZ-182 21 Praha 8, Czech Republic; 4 Department of Physics and Institute for Nanoscience and Engineering, University of Arkansas, Fayetteville, Arkansas 72701, USA

## Abstract

Relaxors are complex materials with unusual properties that have been puzzling the scientific community since their discovery. The main characteristic of relaxors, that is, their dielectric relaxation, remains unclear and is still under debate. The difficulty to conduct measurements at frequencies ranging from ≃1 GHz to ≃1 THz and the challenge of developing models to capture their complex dynamical responses are among the reasons for such a situation. Here, we report first-principles-based molecular dynamic simulations of lead-free Ba(Zr_0.5_Ti_0.5_)O_3_, which allows us to obtain its subterahertz dynamics. This approach reproduces the striking characteristics of relaxors including the dielectric relaxation, the constant-loss behaviour, the diffuse maximum in the temperature dependence of susceptibility, the substantial widening of dielectric spectrum on cooling and the resulting Vogel–Fulcher law. The simulations further relate such features to the decomposed dielectric responses, each associated with its own polarization mechanism, therefore, enhancing the current understanding of relaxor behaviour.

## Abstract (web summary)

Relaxor ferroelectrics possess potential microwave frequency applications due to characteristic dielectric relaxation properties however the underlying mechanism is debated. Here, the authors use first-principles-based molecular dynamic simulations to understand such behaviour in Ba(Zr_0.5_Ti_0.5_)O_3_.

## Graphical abstract

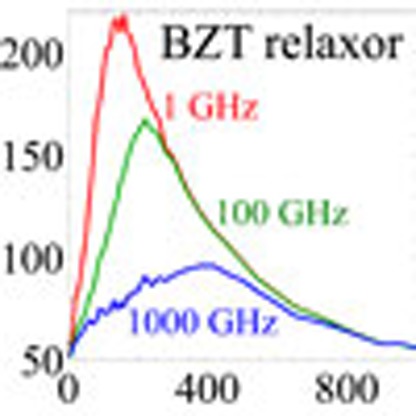

Relaxor ferroelectrics are fascinating materials whose major unusual characteristics are related to dipolar relaxation, hence the name relaxor given to them. Such relaxation manifests itself by the fact that the real part of their dielectric response versus temperature exhibits a broad peak, at *T*=*T*_m_, which is strongly frequency dependent^1^,^2^. The origin of this dipolar relaxation is still open for discussion. A commonly proposed explanation is that it arises from the motion of polar nanoregions (PNRs), which appear on cooling at the so-called Burns temperature, *T*_B_ (refs 3, 4, 5). However, such a widely accepted mechanism has been recently challenged in (lead-based) relaxors on the ground of analysis of measured structural data^6^,^7^,^8^ (note that the controversy also extends to atomistic simulations, since some computational works did report the existence of PNRs in relaxor ferroelectrics^4^,^9^,^10^ while others did not^11^,^12^,^13^). This lack of consensus is largely due to the experimental challenges in observing PNRs directly and measuring some properties they are expected to exhibit, such as the dielectric spectra for frequencies ranging between 10^9^ and 10^12^ Hz. As a matter of fact, while low frequencies (typically between 10^−3^ and 10^9^ Hz) can be accessed by dielectric spectroscopy and high frequencies (typically phonons with frequencies above 10^12^ Hz) by the infrared reflectivity technique^2^,^14^,^15^,^16^,^17^, very little data at limited temperatures are currently available, primarily from time-domain terahertz transmission spectroscopy^15^,^16^, in the intermediate frequency range 10^9^–10^12^ Hz. As a result, details of the relaxation spectrum (and, consequently, the details of the dipolar dynamics) remain unknown at *T*≃*T*_B_, where relaxation exists at subterahertz frequencies only. At lower temperatures, the part of the spectrum where relaxation and phonon dynamics merge, is thus also poorly understood.

This article reports first-principles-based simulations on the lead-free Ba(Zr_0.5_Ti_0.5_)O_3_ (BZT) relaxor ferroelectrics in the subterahertz frequency range that resolve this outstanding problem. Atomistic features of our numerical tool further reveal the role of the Ti- and Zr-centred dipoles, and their cross-correlations in determining some relaxor characteristics such as the Vogel–Fulcher law (VF) obeyed by *T*_m_. Note that BZT is chosen here because BaTiO_3_-based relaxors proved both experimentally^5^,^17^ and theoretically^4^,^18^,^19^,^20^ to exhibit PNRs and because some experimental data on BZT (to test the predictions against) are available at some terahertz and subterahertz frequencies.

## Results

### Dielectric response

As indicated in the Methods section below, molecular dynamics simulations using the effective Hamiltonian of ref. 4 are conducted to predict the complex dielectric response at given temperatures. Practically, a series of molecular dynamics simulations is performed at 50 different temperatures between 5 and 1,000 K. After computing the dielectric response via equation (2) indicated in the Methods section, we analyse the obtained spectrum at each temperature. To this end, we apply an approach that is typically used in experimental dielectric spectroscopy, that is, to fit the data with an analytical expression written as a sum of standard empirical functions, each of which is the contribution of a particular polarization mechanism. We use the following expression (for the average diagonal element of the dielectric tensor):


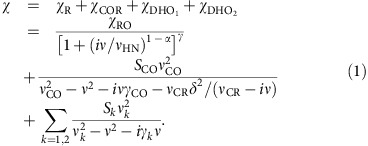


The first term of this sum is the Havriliak–Negami (HN) function, which represents a relaxation mechanism. In this function, *α* and *γ* are parameters characterizing the shape of the relaxation spectrum, and *χ*_RO_ and *ν*_HN_ are the dielectric strength and the characteristic frequency of this relaxation mechanism, respectively. The second term of equation (1) describes a coupled oscillator-relaxator (COR) mechanism, with *S*_CO_, *ν*_CO_, and *γ*_CO_ being the dielectric strength, resonant frequency and damping constant of the coupled oscillator mode, respectively, while *ν*_RO_ is the bare relaxation frequency of the coupled relaxation mode and *δ* is the coupling constant characterizing the strength of interaction between the oscillator and the relaxator. The last two terms represent two damped harmonic oscillators (DHO) of dielectric strength *S*_*k*_, resonant frequency *ν*_*k*_ and damping constant *γ*_*k*_, with *k*=1, 2. Note that, during our nonlinear least square fitting procedure, the real and imaginary parts are fitted simultaneously in the entire frequency range varying between 10^9^ and 2 × 10^13^ Hz (0.033–667 cm^−1^), where meaningful data are available.

**Figure 1:**
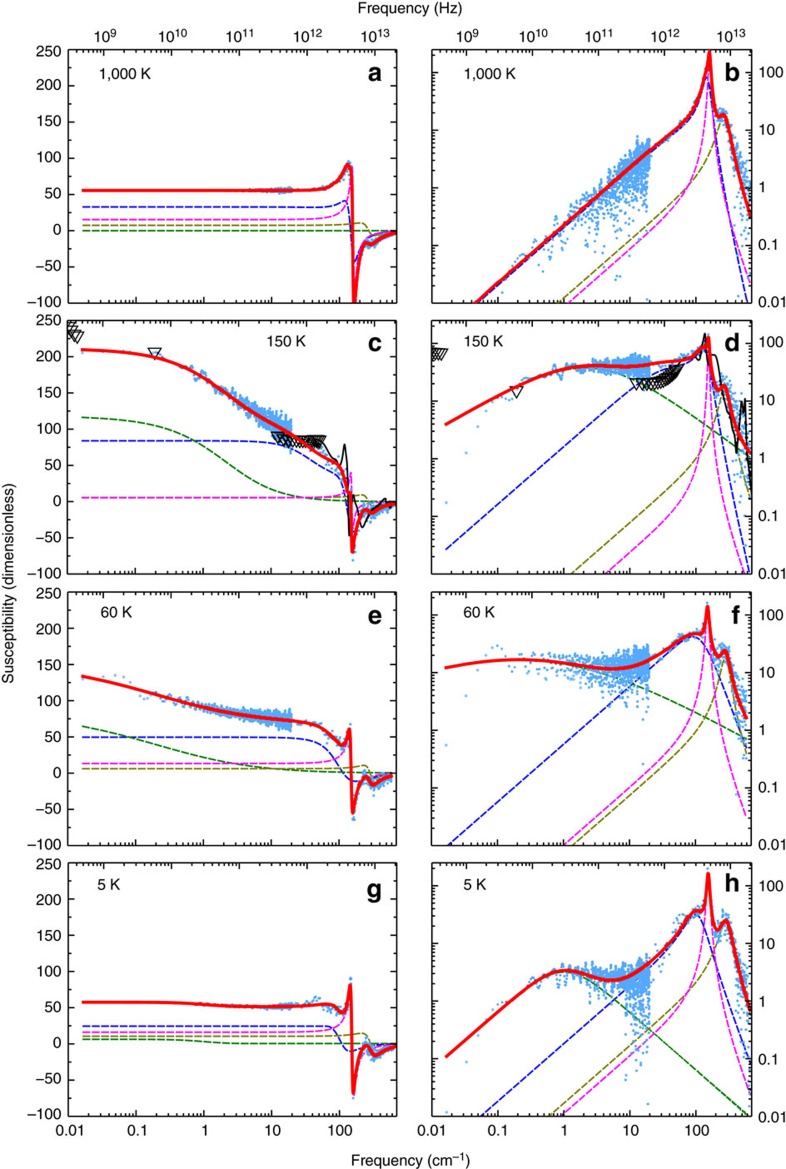
Dielectric relaxation spectra of the compositionally disordered Ba(Zr_0.5_Ti_0.5_)O_3_ crystal at selected temperatures. (**a**–**h**) Frequency dependence of real (**a**,**c**,**e**,**g**) and imaginary (**b**,**d**,**f**,**h**) parts of the dielectric susceptibility at 1,000 K (**a**,**b**), 150 K (**c**,**d**), 60 K (**e**,**f**) and 5 K (**g**,**h**). The blue symbols are the results of molecular dynamics simulations; for the sake of clarity, every point at *v*⪞20 cm^−1^ represents the average value over 100 neighbouring simulation points. The thick red line is the fit to equation (1); green, blue, magenta and olive dashed lines show the contributions of relaxation and oscillation mechanisms, χ_R_, χ_COR_, 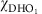 and 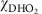, respectively, that comprise the total response. The triangles and thin black lines in **c**,**d** are the experimental data for the ceramic Ba(Zr_0.6_Ti_0.4_)0_3_ of close composition taken from fig. 5 of ref. 16. Note that between 0.01 and 7 cm^−1^ only one experimental point (at 0.2 cm^−1^) is available, which makes it impossible to determine reliably the parameters of the relaxation process based on experimental data.

The simulated spectra and the results of their fitting to equation (1) are shown for selected temperatures in Fig. 1 (fitting results at some other temperatures can be found in the Supplementary Movie 1). One can see that, in all cases, the isothermal frequency dependence of the complex total susceptibility can be well described by equation (1), which justifies the choice of such an equation. Figure 1 further provides the decomposition of *χ* into four separate terms of equation (1), and thereby demonstrates that *χ*_R_ is the dominant effect for the lowest frequencies, while *χ*_COR_ and the DHOs contribute to the dielectric response for the intermediate and highest frequencies, respectively. Note that, at microscopic level, our simulations reveal that the intermediate DHO_1_ mode is mostly related to the phonon vibration of the Zr ions, while the DHO_2_ mode is associated with the high-frequency phonon vibrations of the Ti ions, which is consistent with ref. 20. The COR mechanism corresponds to a mixed relaxation-oscillation mode within the Ti subsystem, that is a single Debye relaxation mode coupled to an optical phonon mode, while the HN function corresponds to a more complex relaxation mode arising from the influence of different chemical environments on Ti-centred dipoles. Moreover, Fig. 1c,d also display the experimental data at 150 K of ref. 16 by means of triangles. The agreement between the simulations and the measured data is quite remarkable for both the real and imaginary parts of the dielectric response, especially given that Fig. 1 shows that these two parts are very sensitive to temperature. Such an agreement asserts the validity and accuracy of the present simulations.

### Relaxation

**Figure 2:**
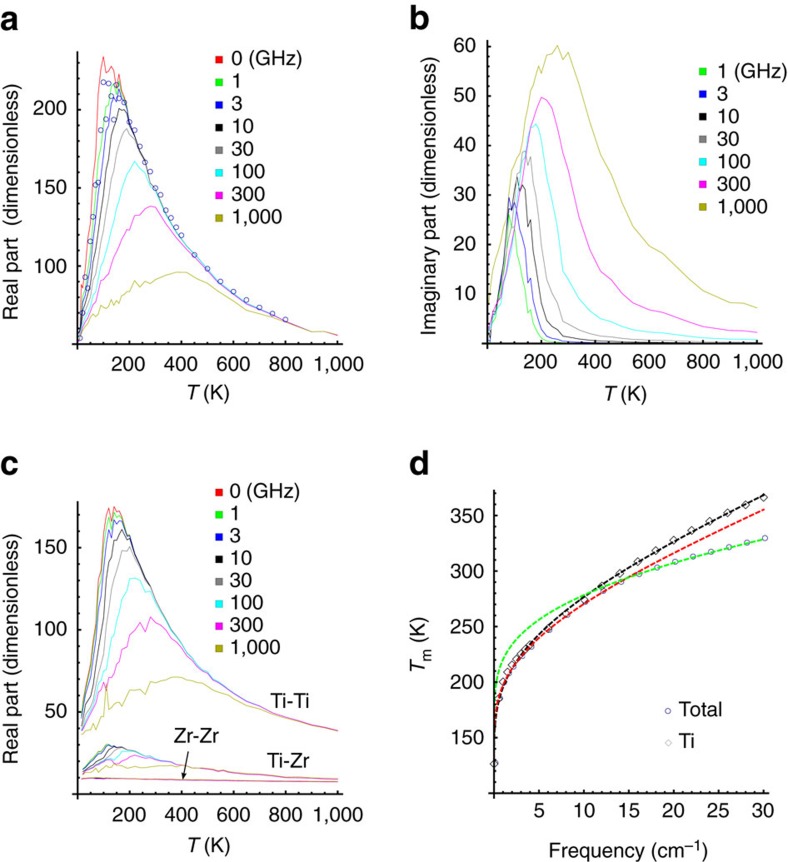
Temperature dependences of the complex susceptibility for different probing frequencies and the fitting to the VF law. (**a**) Real part of the total susceptibility. Results obtained from previous Monte–Carlo method simulations^4^ are also shown as blue circles. (**b**) Imaginary part of the total susceptibility. (**c**) Different contributions to the real part of total susceptibility (from top to bottom): *χ*^Ti,Ti^, *χ*^Ti,Zr^, *χ*^Zr,Zr^. (**d**) Frequency dependence of the temperatures at which the real parts of *χ* and *χ*^Ti,Ti^ are maximum (hollow circles and diamonds, respectively), with the solid lines fitting these curves to the VF laws.

Having demonstrated the accuracy of our simulation, let us now use the best-fit relaxation parameters of equation (1) to calculate *χ*(*T*, *ν*) at selected temperatures and probing frequencies. The resulting real and imaginary parts of this total dielectric response as a function of temperature are reported in Fig. 2a,b, respectively, for different frequencies ranging from 1 GHz to 1 THz. One can see that both the real and imaginary parts of the predicted dielectric response exhibit a broad peak over temperature, which shifts in position when the probing frequency changes. In other words, our present first-principles-derived simulations reproduce the main characteristics (dielectric dispersion) of relaxor ferroelectrics. Moreover, Fig. 2b also reveals that the simulated imaginary part of the dielectric response is practically insensitive to frequency for low temperatures (below ∼150 K), which is experimentally known to occur in relaxor ferroelectrics^21^,^22^, and is often referred to as the constant-loss behaviour.

Figure 2a also displays the dielectric response obtained from the extrapolation of our numerical molecular dynamics data down to zero frequency, that is the dielectric response given by *χ*_RO_+*χ*_COR_(*ν*=0)+*S*_1_+*S*_2_, along with the dielectric response obtained from our previous effective Hamiltonian simulations using the Monte–Carlo method^4^. Interestingly, as evidenced in Fig. 2a, such two static responses agree very well with each other, which further supports the scheme described by equation (1) to fit the molecular dynamics outputs. In particular, these two responses are found to possess a maximum at some finite temperature (of the order of 100 K), similar to the peak experimentally found for the static susceptibility in some lead-based and lead-free relaxors^2^,^22^,^23^.

### Fitting parameters

**Figure 3:**
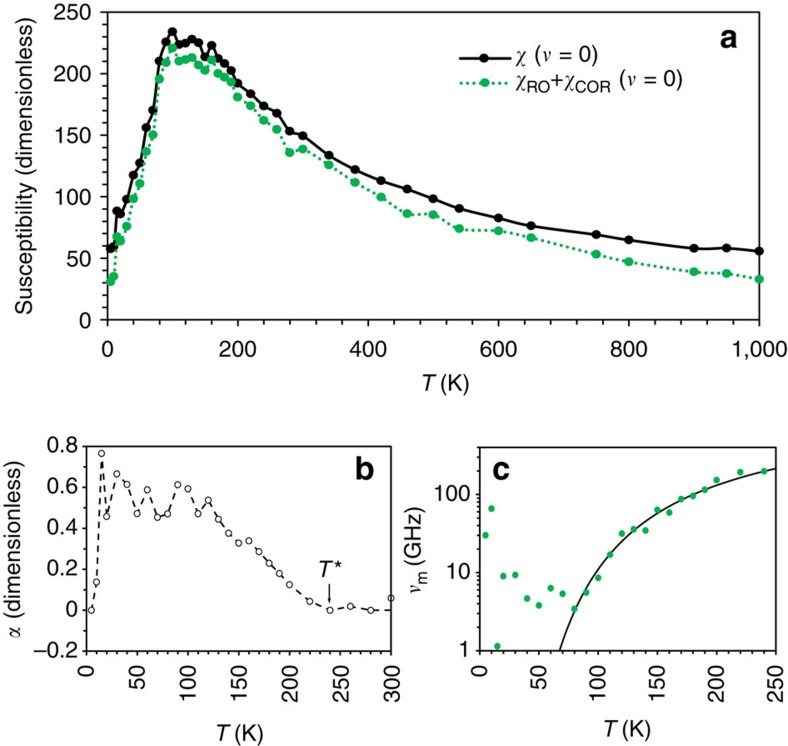
Parameters of the dielectric spectra in the compositionally disordered Ba(Zr_0.5_Ti_0.5_)O_3_ crystal as functions of temperature. (**a**) Total static susceptibility *χ* (*v*=0), and static susceptibility related to all polarization processes in which relaxation is involved, *χ*_RO_+*χ*_C0R_ (*v*=0). (**b**,**c**) Parameters of the HN relaxation process: the parameter *α*, characterizing the width of relaxation spectrum (**b**) and the most probable relaxation frequency, *v*_m_ (**c**). The dashed line in **b** is the guide to the eyes and the solid line in **c** is the fit to the Arrhenius law.

To gain a deeper insight into the results depicted in Fig. 2a,b, we take a close look at the temperature dependences of some parameters appearing in equation (1). Such dependences are shown in Fig. 3. More precisely, Fig. 3a reports the total static susceptibility, *χ*(*ν*=0), associated with equation (1), as well as its separate contributions from the mechanisms involving relaxation, that is, *χ*_RO_+*χ*_COR_(*ν*=0). Figure 3b displays the *α* parameter associated with the HN function (equation (1)), while Fig. 3c shows the frequency, *ν*_m_, at which Im*χ*_R_(*ν*) peaks for any given temperature (*ν*_m_ is known as the most probable relaxation frequency and may differ from *ν*_HN_). The Supplementary Fig. 1 and Supplementary Note 1 provide information about other parameters not shown in Fig. 3. It is clear from Fig. 3a that, for temperatures above the well-known Burns temperature *T*_B_≃450 K of this system^4^,^17^, the total static dielectric susceptibility is dominated by the relaxation mechanisms, or, equivalently, that the DHOs contribute only very slightly to it (the contribution from DHOs is the difference between the black and green curves of Fig. 3a, which is found to be ∼20 for any investigated temperature). Such features contrast with the case of lead-based perovskite relaxors, where the response is purely of phonon (DHO) nature for *T*>*T*_B_ and relaxation appears only at *T*<*T*_B_ (refs 24, 25). The large and small contributions of the relaxation mechanisms and the DHOs, respectively, result in the Curie–Weiss law behaviour for *T*>*T*_*B*_ with a negative critical temperature, *T*_CW_, in agreement with the Monte–Carlo method simulations^4^ and experimental results^17^. Moreover, *χ*_RO_+*χ*_COR_(*ν*=0) exhibits a peak at *T*≃100 K, which is found to be fully responsible for the maximum in the temperature dependence of the total static susceptibility reported in Fig. 2a (note also that the broadness aspect of the total susceptibility is found to originate from the temperature behaviour of the static contributions of the relaxation mechanisms). Supplementary Fig. 1 further shows the separate contribution of *χ*_COR_(*ν*=0) and *χ*_RO_ as a function of temperature. In particular, *χ*_RO_ is found to practically vanish for temperatures above *T*_B_ (implying that the COR mechanism is the most predominant player there), while *χ*_RO_ is about twice as big as *χ*_COR_(*ν*=0) for temperatures close to 100 K. However, we numerically found that the HN and COR contributions significantly overlap in frequency, especially at comparatively high temperatures. As a result, some of the parameters of these two contributions can only be estimated rather than reliably determined. One should therefore consider the temperature dependence of the sum of *χ*_RO_ and *χ*_COR_ rather than look at them separately. This explains why this sum is shown in Fig. 3a.

It is also important to realize that Fig. 3b reveals that the *α* parameter departs from zero at the temperature of ≃240 K, that is, at the so-called T* characteristic temperature of BZT, below which Ti-rich PNRs were found to begin interacting with each other in an antiferroelectric-like fashion in ref. 4. This departure from zero may indicate the existence of a distribution of Debye modes with different relaxation times. *α* then increases up to ≃0.5 when cooling the system from T* to ≃130 K. It remains quite large when further decreasing the temperature down to 5–10 K, which results in frequency broadening of the Im*χ*_R_(*ν*) peak. In fact, the peak becomes so wide at temperatures below ≃100 K (Fig. 1f) that the loss is practically constant with frequency (cf. Fig. 2b). Note that the broadening of relaxation spectrum on cooling and the constant-loss behaviour are two characteristic features of relaxor ferroelectrics, in contrast to the behaviour of most other dielectrics where the shape of the spectrum is temperature independent^26^. Figure 3b,c also indicate that the extracted *α* and *ν*_m_ parameters exhibit some scattering for temperatures ranging between ≃15 and 80 K. This is because the characteristic relaxation frequency, *ν*_m_, reaches the low-frequency boundary of the available molecular dynamics data in that temperature range (as shown in Fig. 3c) and, as a result, the HN parameters become uncertain. On the other hand, at the very low temperatures of 5–10 K, the relaxation response moves back to the simulation frequency window, as evidenced in Fig. 1g,h, which allows us to precisely extract again the values of *α*. Interestingly, this parameter is now very close to zero as revealed by Fig. 3b, which explains why the loss peak is now narrower (cf. Fig. 1h) and which is characteristic of a simple Debye (exponential in time domain) relaxation. Note that the remaining relaxation at this temperature is numerically found to be caused by dipoles associated with Ti ions surrounded mostly by Zr ions (not shown here).

### Arrhenius and VF laws

Let us now discuss the temperature dependence of *ν*_m_ depicted in Fig. 3c. Fitting this relation for temperatures above 70 K with the VF law, *ν*_m_=*ν*_a_ exp[−*E*_a_/*k*_B_(*T*−*T*_0_)] shows that *T*_0_=(−2±9) K, that is, *T*_0_ can be safely assumed to vanish. In other words, *ν*_m_ rather follows an Arrhenius law *ν*_m_=*ν*_a_ exp(−*E*_a_/*k*_B_*T*) above ≃70 K, with the best-fit parameters *ν*_a_=1.6 × 10^12^ Hz (53 cm^−1^) and *E*_a_=0.043 eV. Interestingly, an Arrhenius law having a similar *ν*_a_, but four times larger *E*_a_, has been experimentally found in BZT (refs 15, 16) for a different, lower-in-frequency relaxation mode. The fact that both the present simulations and the experiments indicate that *T*_0_ vanishes (that is, it is not finite) in BZT can be understood in terms of the semi-phenomenological model proposed by Pirc and Blinc^27^. This model suggests that *T*_0_ corresponds to the temperature at which the PNRs grow into an infinite cluster as temperature decreases, which explains why some relaxor systems exhibit a VF law behaviour. Such a scenario is relevant to lead-based relaxors according to a recent study^28^. However, PNRs in BZT are confined to Ti-rich regions and cannot grow into an infinite cluster with decreasing temperature (cf. ref. 4) because these PNRs are immersed into a Zr-rich matrix that is only weakly polarizable. Consistent with the fact that Pirc and Blinc have shown that the VF law would reduce to the Arrhenius law if PNRs did not grow, we can conclude that the Arrhenius law, rather than the VF law, applies to BZT. Note that Fig. 3c further shows that the Arrhenius law is not obeyed at lower temperatures, which is consistent with ref. 20.

Furthermore, the data displayed in Fig. 2a also allow us to obtain the temperature, *T*_m_, at which the real part of the dielectric response peaks, for any probing frequency, *ν* (the *T*_m_ versus *ν* for the imaginary part of the susceptibility is shown in Supplementary Fig. 2 and discussed in Supplementary Note 2). The dependence of *T*_m_ on *ν* is reported in Fig. 2d, and it neither follows Arrhenius behaviour nor a single VF relation. In fact, Fig. 2d shows that *T*_m_ obeys two different VF laws of the form *ν*=*ν*_0_ exp[−*U*/(*T*_m_−*T*_VF_)]: one for temperatures below ≃280 K (with the best-fit parameters *ν*_0_=322 cm^−1^, *U*=637 K and *T*_VF_=87 K) and one for temperatures above it (*ν*_0_=1,950 cm^−1^, *U*=1,005 K and *T*_VF_=88 K). These two VF laws have significantly different *U* and *ν*_0_, but similar *T*_VF_≃90 K. Interestingly, this temperature is consistent with the finding in ref. 4 that the thermally activated reorientations of the (Ti-based) dipoles inside the existing PNRs are numerically found to be frozen in BZT below ≃90 K. This *T*_VF_ is also close to the temperature of the static susceptibility maximum shown in Figs 2a and 3a, as predicted by the phenomenological theory of VF relationship for relaxors proposed by Tagantsev^29^. In particular, it is interesting to realize that refs 22, 29 indicate that *T*_m_ can follow a VF law even if the characteristic relaxation frequency obeys the Arrhenius behaviour provided that the static dielectric response possesses a maximum at a finite temperature, which is consistent with the present data shown in Fig. 2d (for *T*_m_), Fig. 3c (for *ν*_m_) and Fig. 2a (for the static dielectric response). Note that Tagantsev's model predicts the fulfillment of a single VF law for the *T*_m_(*ν*) values belonging to the temperature interval above *T*_m_(*ν*=0), but neither the size of this interval nor the *T*_m_(*ν*) behaviour at higher temperatures are specified (they are determined by several factors including the shape of temperature dependence of static susceptibility, the temperature dependence of characteristic times of the contributing relaxation modes, and the shape of relaxation spectrum^22^). In fact, two different VF laws have been observed experimentally in some relaxors^30^, with the second VF law occurring in a higher temperature range that cannot be described by Tagantsev's model.

It is also worth emphasizing that, in Tagantsev's model for *T*_m_, *U* is not an activation energy and *T*_VF_ is not the temperature where the relaxation frequency vanishes, that is, *T*_VF_ could be different from *T*_0_ (the critical temperature involved in the temperature behaviour of *ν*_m_), while in canonical lead-based relaxors these two temperatures are experimentally found to be close to each other. Therefore, like in other relaxors, the dielectric response in BZT differs fundamentally from that in ideal paraelectrics, where the static susceptibility and the characteristic relaxation time diverge simultaneously (in proportion to 1/*T*) as the temperature approaches zero (ref. 26). However, in canonical lead-containing relaxors the relaxation time diverges at the characteristic VF temperature *T*_0_>0 (ref. 21), while, in BZT, the relaxation time remains finite at *T*>0, but the dielectric strength (static susceptibility) of relaxation mode peaks at another characteristic temperature, *T*_m_(*v*=0)≃*T*_VF_, and tends towards zero on further cooling.

Note also that a detailed analysis (Supplementary Fig. 3 and Supplementary Note 3) shows that the two relaxation-related modes (HN and COR), we have predicted in our simulations, are also seen in experiments (they are traditionally called central modes). However, their behaviour cannot be accurately verified because of insufficiency of experimental data. Moreover, experiments also report an additional relaxation mode in BZT which dominates at very low frequency (*ν*<1 GHz). This latter mode cannot be mimicked by our present simulations and was suggested to originate from the collective dynamics inside PNRs or the thermally activated reorientations of the PNRs (ref. 20).

### Atomistic decomposition

To elucidate further the origin of the dielectric response in BZT and to better understand the existence of two VF laws depicted in Fig. 2d, we take advantage of another strength provided by our atomistic simulations, that is the possibility to separate the total dielectric response into its constituent parts: the *χ*^Ti,Ti^ contribution from the 5-atom cells centred on Ti sites, the *χ*^Zr,Zr^ contribution from the 5-atom cells centred on Zr sites and the *χ*^Ti,Zr^ correlation between these two different types of 5-atom cells. This decomposition of the total dielectric response is (unfortunately) impossible to obtain in experiments but is made feasible here, thanks to the previously developed technique utilizing, in equation (2), the fact that **d**=**d**^Ti^+**d**^Zr^, where **d** is the total electric dipole moment of the whole system, while **d**^Ti^ and **d**^Zr^ are the electric dipole moments of the 5-atom cells centred on the Ti and Zr ions, respectively^20^ [note that the effective Hamiltonian used here^4^ possesses the so-called local modes as degrees of freedom. These local modes are technically centred on the B-sites (Ti or Zr), represent the collective displacements of the Ba, B and oxygen ions within any 5-atom unit cell, and are directly proportional to the electric dipole moments of each 5-atom cell]. Figure 2c shows the resulting real parts of *χ*^Ti,Ti^, *χ*^Ti,Zr^ and *χ*^Zr,Zr^ as a function of temperature, for different probing frequencies. The real part of *χ*^Ti,Ti^ resembles the total susceptibility of Fig. 2a in every aspect except that its maximum value is ∼20% smaller. In contrast, the real parts of *χ*^Ti,Zr^ and *χ*^Zr,Zr^ are much smaller in both magnitude (approximately one tenth of *χ*^Ti,Ti^) and dispersion. In particular, the susceptibility of *χ*^Zr,Zr^ is essentially a constant over temperatures and frequencies. These results therefore indicate that the total dielectric response of disordered BZT and its accompanying dispersion are mainly dominated by the response arising from the Ti-centred 5-atom cells, which is consistent with our aforementioned finding that the HN and COR mechanisms (which concern the Ti subsystem) are mostly responsible for the unusual dielectric features of BZT below *T*_B_. Interestingly, one can also extract from Fig. 2c the temperature *T*_m,Ti_ at which the real part of *χ*^Ti,Ti^ peaks for any probing frequency. The resulting *T*_m,Ti_-versus-*ν* curve is also reported in Fig. 2d, and can be well fitted at any temperature by a single VF law, with the best-fit parameters *ν*_0_=361 cm^−1^, *U*=745 K and *T*_VF_=69 K. It is striking to realize that the *T*_m,Ti_(*ν*) function is virtually identical to that of the total response for any temperature ≲240 K (which is, incidentally, T*), but differs for the temperatures above. For these higher temperatures, one can see in Fig. 2c that the real part of *χ*^Ti,Zr^ disperses much less than that of *χ*^Ti,Ti^, which leads to a reduction of *T*_m_ with respect to *T*_m,Ti_ above 280 K.

## Discussion

In summary, we report first-principles-derived simulations in BZT that reproduce the main characteristics of relaxor ferroelectrics, that is the frequency dependence of the real and imaginary parts of the dielectric response versus temperature. Analysis of these simulated results reveals that the relaxation-type polarization processes dominate in the dielectric response of disordered BZT for all temperatures, which contrasts with lead-based perovskite relaxors in which the response is purely of phonon nature for *T*>*T*_*B*_ and the relaxation only appears below *T*_B_ (refs 24, 25). Such a dramatic difference may originate from the fact that BZT has been reported to exhibit PNRs below *T*_B_ (refs 4, 5, 18, 19, 20), while the PNRs as compact regions of ferroelectric order might not exist inside a nonpolar matrix in the classical lead magnesium niobate relaxor according to recent diffuse neutron and x-ray scattering^6^,^7^ and x-ray fluorescence holography experiments^8^. Furthermore, the significance of the characteristic relaxor temperature T* of BZT in terms of dipolar relaxation is also clearly determined by the present study: above this temperature, the relaxation consists of a single Debye mode (corresponding to *α*=0) coupled to an optical phonon mode within the Ti subsystem, while below T*, the Ti-centred dipoles subject to different chemical environments result in a relaxation spectrum that significantly broadens (*α*>0), signifying the distribution of Debye relaxation times. The present computational results also predict that the frequency dependence of the *T*_m_ temperature at which the real part of the total dielectric response peaks is well described (below ≃280 K) by a single VF law. The second VF law having a different parameter *U* but identical *T*_VF_ is further found to describe the behaviour of *T*_m_ versus frequency for the temperatures above ≃280 K. We therefore believe that the present study deepens our knowledge on relaxor ferroelectrics, in general, and their characteristic dipolar relaxations, in particular, which are responsible for their unusual properties.

## Methods

### First-principles model

We employ the first-principles-based effective Hamiltonian that has been developed in ref. 4 and successfully used to model and study different static and dynamical properties of the BZT systems^18^,^19^,^20^. This Hamiltonian is presently implemented within the molecular dynamics technique described in refs 20, 31, 32, 33, 34, 35, and is applied to a 12 × 12 × 12 supercell made from disordered Ba(Zr_0.5_Ti_0.5_)O_3_ solid solution. The complex dielectric susceptibility, *χ*_*lm*_(*ν*), can be obtained from the molecular dynamics simulations via refs 20, 31, 33, 36, 37:


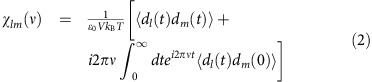


where *ν* is the frequency, while *l* and *m* define Cartesian components, *V* is the volume of the chosen supercell, *ɛ*_0_ is the vacuum permittivity and **d**(*t*) is the electric dipole moment of the system at time *t*, and 〈...〉 represents thermal averages. At any given time *t*, **d**(*t*) is obtained by summing over every dipole of the system, and therefore contains contributions from different compositional regions that have different dipole dynamics, including relaxational dynamics. Note the important technical differences with respect to previous studies^20^,^32^,^33^,^34^,^35^,^38^,^39^: the total molecular dynamics simulation time is extended to 16 ns, which is about eight times longer than the simulation time used in refs 20, 32, 33, 34, 35, 38, 39, to accurately mimic the low-frequency part of the dielectric response; and the real part of the dielectric response is presently investigated in detail, in addition to the imaginary part.

## Additional information

**How to cite this article:** Wang, D. *et al*. Subterahertz dielectric relaxation in lead-free Ba(Zr,Ti)O_3_ relaxor ferroelectrics. *Nat. Commun.* 7:11014 doi: 10.1038/ncomms11014 (2016).

## Supplementary Material

Supplementary Information Supplementary Figures 1-3, Supplementary Notes 1-3 and Supplementary References

Supplementary Movie 1 This video shows the fitting of the simulated dielectric response using Eq. (1) at various temperatures.

## References

[b1] Colla E., Koroleva E., Okuneva N. & Vakhrushev S. Low-frequency dielectric response of PbMg_1/3_Nb_2/3_O_3_. J. Phys. Condens. Matter 4, 3671–3677 (1992).

[b2] Bokov A. A., Maglione M. & Ye Z.-G. Quasi-ferroelectric state in Ba(Ti_1−*x*_Zr_*x*_)O_3_ relaxor: dielectric spectroscopy evidence. J. Phys. Condens. Matter 19, 092001 (2007).

[b3] Hiraka H., Lee S., Gehring P., Xu G. & Shirane G. Cold neutron study on the diffuse scattering and phonon excitations in the relaxor Pb(Mg_1/3_Nb_2/3_)O_3_. Phys. Rev. B 70, 184105 (2004).

[b4] Akbarzadeh A., Prosandeev S., Walter E., Al-Barakaty A. & Bellaiche L. Finite-temperature properties of Ba(Zr,Ti)O_3_ relaxors from first principles. Phys. Rev. Lett. 108, 257601 (2012). 23004657 10.1103/PhysRevLett.108.257601

[b5] Xie L. . Static and dynamic polar nanoregions in relaxor ferroelectric Ba(Ti_1−*x*_,Sn_*x*_)O_3_ system at high temperature. Phys. Rev. B 85, 014118 (2012).

[b6] Bosak A., Chernyshov D. & Krisch M. Diffuse scattering in relaxor ferroelectrics: true three-dimensional mapping, experimental artefacts and modelling. Acta Crystallogr. A 68, 117–123 (2012). 22186288 10.1107/S0108767311040281

[b7] Hlinka J. Do we need the ether of polar nanoregions? J. Adv. Dielectr. 2, 1241006 (2012).

[b8] Hu W. . Acute and obtuse rhombohedrons in the local structures of relaxor ferroelectric Pb(Mg_1/3_Nb_2/3_)O_3_. Phys. Rev. B 89, 140103 (R) (2014).

[b9] Tinte S., Burton B. P., Cockayne E. & Waghmare U. V. Origin of the relaxor state in Pb(B_*x*_B′_1−*x*_)O_3_ perovskites. Phys. Rev. Lett. 97, 137601 (2006). 17026074 10.1103/PhysRevLett.97.137601

[b10] Paściak M., Welberry T. R., Kulda J., Kempa M. & Hlinka J. Polar nanoregions and diffuse scattering in the relaxor ferroelectric Pb(Mg_1/3_Nb_2/3_)O_3_. Phys. Rev. B 85, 224109 (2012).

[b11] Takenaka H., Grinberg I. & Rappe A. M. Anisotropic local correlations and dynamics in a relaxor ferroelectric. Phys. Rev. Lett. 110, 147602 (2013). 25167037 10.1103/PhysRevLett.110.147602

[b12] Grinberg I., Shin Y.-H. & Rappe A. M. Molecular dynamics study of dielectric response in a relaxor ferroelectric. Phys. Rev. Lett. 103, 197601 (2009). 20365954 10.1103/PhysRevLett.103.197601

[b13] Sepliarsky M. & Cohen R. E. First-principles based atomistic modeling of phase stability in PMN-*x*PT. J. Phys. Condens. Matter 23, 435902 (2011). 21997277 10.1088/0953-8984/23/43/435902

[b14] Ke S., Fan H., Huang H., Chan H. & Yu S. Dielectric dispersion behavior of Ba(Zr_*x*_Ti_1−*x*_)O_3_ solid solutions with a quasiferroelectric state. J. Appl. Phys. 104, 034108 (2008).

[b15] Nuzhnyy D. . Broadband dielectric response of Ba(Zr,Ti)O_3_ ceramics: from incipient via relaxor and diffuse up to classical ferroelectric behavior. Phys. Rev. B 86, 014106 (2012).

[b16] Petzelt J. . Broadband dielectric spectroscopy of Ba(Zr,Ti)O_3_: dynamics of relaxors and diffuse ferroelectrics. Ferroelectrics 469, 14–25 (2014).

[b17] Maiti T., Guo R. & Bhalla A. Structure-property phase diagram of Ba(Zr,Ti)O_3_ system. J. Am. Ceram. Soc. 91, 1769–1780 (2008).

[b18] Prosandeev S., Wang D., Akbarzadeh A., Dkhil B. & Bellaiche L. Field-induced percolation of polar nanoregions in relaxor ferroelectrics. Phys. Rev. Lett. 110, 207601 (2013). 25167451 10.1103/PhysRevLett.110.207601

[b19] Prosandeev S., Wang D. & Bellaiche L. Properties of epitaxial films made of relaxor ferroelectrics. Phys. Rev. Lett. 111, 247602 (2013). 24483699 10.1103/PhysRevLett.111.247602

[b20] Wang D. . Fano resonance and dipolar relaxation in lead-free relaxors. Nat. Commun. 5, 5100 (2014). 25369904 10.1038/ncomms6100

[b21] Bokov A. A. & Ye Z.-G. Recent progress in relaxor ferroelectrics with perovskite structure. J. Mater. Sci. 41, 31–52 (2006).

[b22] Bokov A. A. & Ye Z.-G. Dielectric relaxation in relaxor ferroelectrics. J. Adv. Dielectr. 2, 1241010 (2012).

[b23] Bokov A. A. & Ye Z.-G. Double freezing of dielectric response in relaxor Pb(Mg_1/3_Nb_2/3_)O_3_ crystals. Phys. Rev. B 74, 132102 (2006).

[b24] Bovtun V. . Broad-band dielectric response of PbMg_1/3_Nb_2/3_O_3_ relaxor ferroelectrics: Single crystals, ceramics and thin films. J. Eur. Ceram. Soc. 26, 2867–2875 (2006).

[b25] Gehring P. Neutron diffuse scattering in lead-based relaxor ferroelectrics and its relationship to the ultra-high piezoelectricity. J. Adv. Dielectr. 2, 1241005 (2012).

[b26] Jonscher A. Dielectric Relaxation in Solids Chelsea Dielectrics Press (1983).

[b27] Pirc R. & Blinc R. Vogel-Fulcher freezing in relaxor ferroelectrics. Phys. Rev. B 76, 020101 (R) (2007).

[b28] Al-Barakaty A., Prosandeev S., Wang D., Dkhil B. & Bellaiche L. Finite-temperature properties of the relaxor PbMg_1/3_Nb_2/3_O_3_ from atomistic simulations. Phys. Rev. B 91, 214117 (2015).

[b29] Tagantsev A. Vogel-Fulcher relationship for the dielectric permittivity of relaxor ferroelectrics. Phys. Rev. Lett. 72, 1100–1103 (1994). 10056617 10.1103/PhysRevLett.72.1100

[b30] Bokov A. A., Leshchenko M., Malitskaya M. & Raevski I. Dielectric spectra and Vogel-Fulcher scaling in Pb(In_0.5_,Nb_0.5_)O_3_ relaxor ferroelectric. J. Phys. Conden. Matter 11, 4899–4911 (1999).

[b31] Ponomareva I., Bellaiche L., Ostapchuk T., Hlinka J. & Petzelt J. Terahertz dielectric response of cubic BaTiO_3_. Phys. Rev. B 77, 012102 (2008). 10.1103/PhysRevLett.101.167402 18999713

[b32] Wang D., Weerasinghe J., Bellaiche L. & Hlinka J. Dynamical coupling in Pb(Zr,Ti)O_3_ solid solutions from first principles. Phys. Rev. B 83, 020301 (R) (2011).

[b33] Wang D., Weerasinghe J., Al-barakati A. & Bellaiche L. Terahertz dielectric response and coupled dynamics of ferroelectrics and multiferroics from effective Hamiltonian simulations. Int. J. Mod. Phys. B 27, 1330016 (2013).

[b34] Weerasinghe J., Wang D. & Bellaiche L. Low-frequency coupled modes in disordered Pb(Zr,Ti)O_3_ solid solutions from first principles. Phys. Rev. B 85, 014301 (2012).

[b35] Weerasinghe J., Wang D. & Bellaiche L. Effect of central mode on the dielectric tunability of ferroelectrics near room temperature: a first-principle-based study. J. Phys. Condens. Matter 25, 252202 (2013). 23719129 10.1088/0953-8984/25/25/252202

[b36] Caillol J., Levesque D. & Weis J. Theoretical calculation of ionic solution properties. J. Chem. Phys. 85, 6645–6657 (1986).

[b37] Hlinka J. . Coexistence of the phonon and relaxation soft modes in the terahertz dielectric response of tetragonal BaTiO_3_. Phys. Rev. Lett. 101, 167402 (2008). 18999713 10.1103/PhysRevLett.101.167402

[b38] Wang D. . Fermi resonance involving nonlinear dynamical couplings in Pb(Zr,Ti)O_3_ solid solutions. Phys. Rev. Lett. 107, 175502 (2011). 22107534 10.1103/PhysRevLett.107.175502

[b39] Wang D., Weerasinghe J. & Bellaiche L. Atomistic molecular dynamic simulations of multiferroics. Phys. Rev. Lett. 109, 067203 (2012). 23006300 10.1103/PhysRevLett.109.067203

