# Supplementary material for: Subterahertz dielectric relaxation in lead-free Ba(Zr,Ti)O3 relaxor ferroelectrics
Source: Nat Commun. 2016 Apr 4;7:11014. doi: 10.1038/ncomms11014 (PMC4822000; doi:10.1038/ncomms11014)
Supplement: Supplementary Information — Supplementary Figures 1-3, Supplementary Notes 1-3 and Supplementary References [file ncomms11014-s1.pdf]

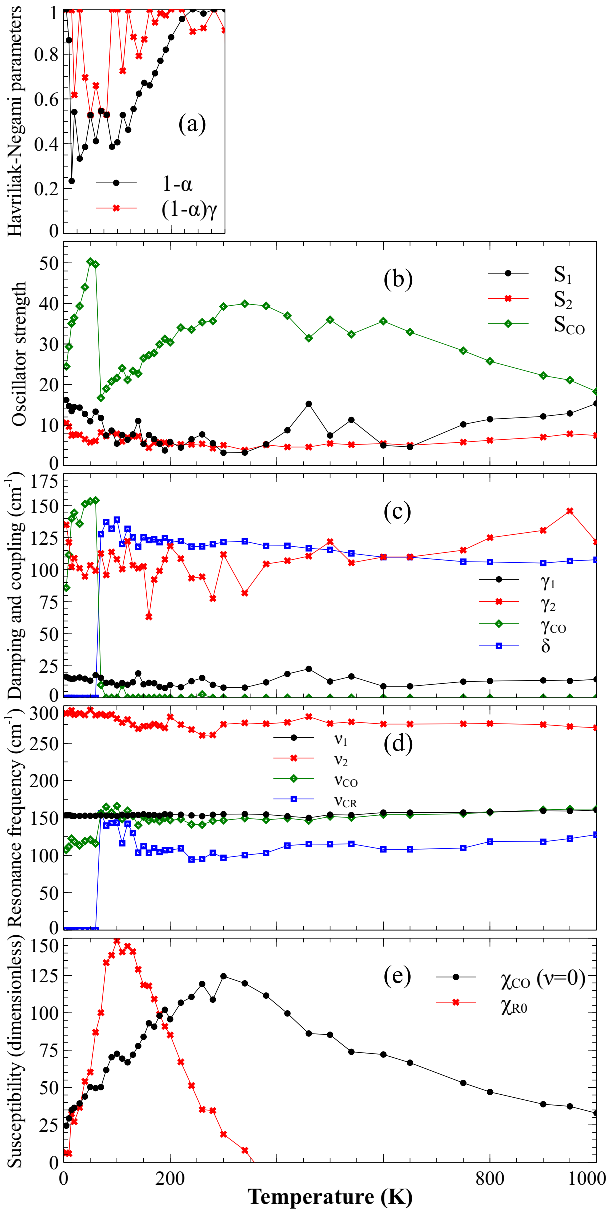

Supplementary Figure 1. Temperature dependence of the fitting parameters appearing in Eq. (1) of the main text. Panel (a) shows α and $(1-\alpha)\,\gamma$ coefficients that are related to the Havriliak-Negami relaxation function [the first term of Eq. (1)]. Panel (b) displays the oscillator strength of the two damped harmonic oscillators (DHOs) [the last term of Eq. (1)] and of the coupled oscillator-relaxator (COR) [the second term of Eq. (1)]. Panel (c) reports the damping constants of these DHOs and COR, as well as the coupling constant, δ, of the COR. Panel (d) shows the resonant frequencies of these DHOs and COR. Panel (e) shows contributions to the static susceptibility from the HN relaxation, χ_{R0}, and the COR mechanism, χ_{COR}.

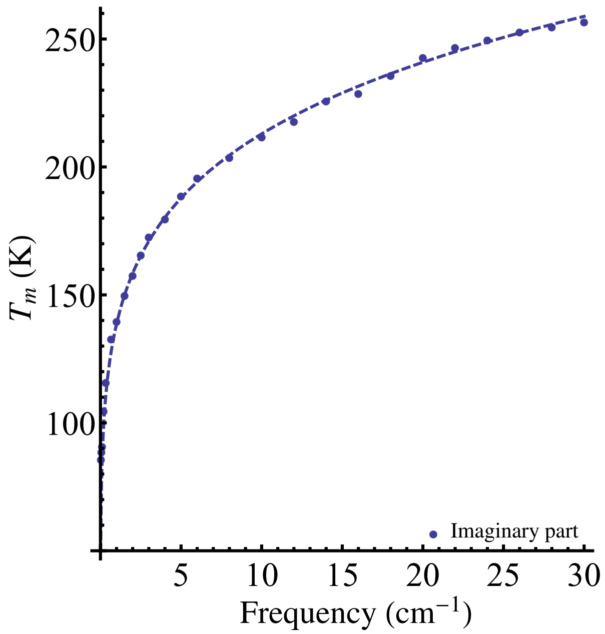

Supplementary Figure 2. The dependence of T_m on frequency. The temperature, T_m, at which the imaginary part of the susceptibility peaks, as a function of the probing frequency. The data points are fitted with the Vogel-Fulcher law (dashed curve).

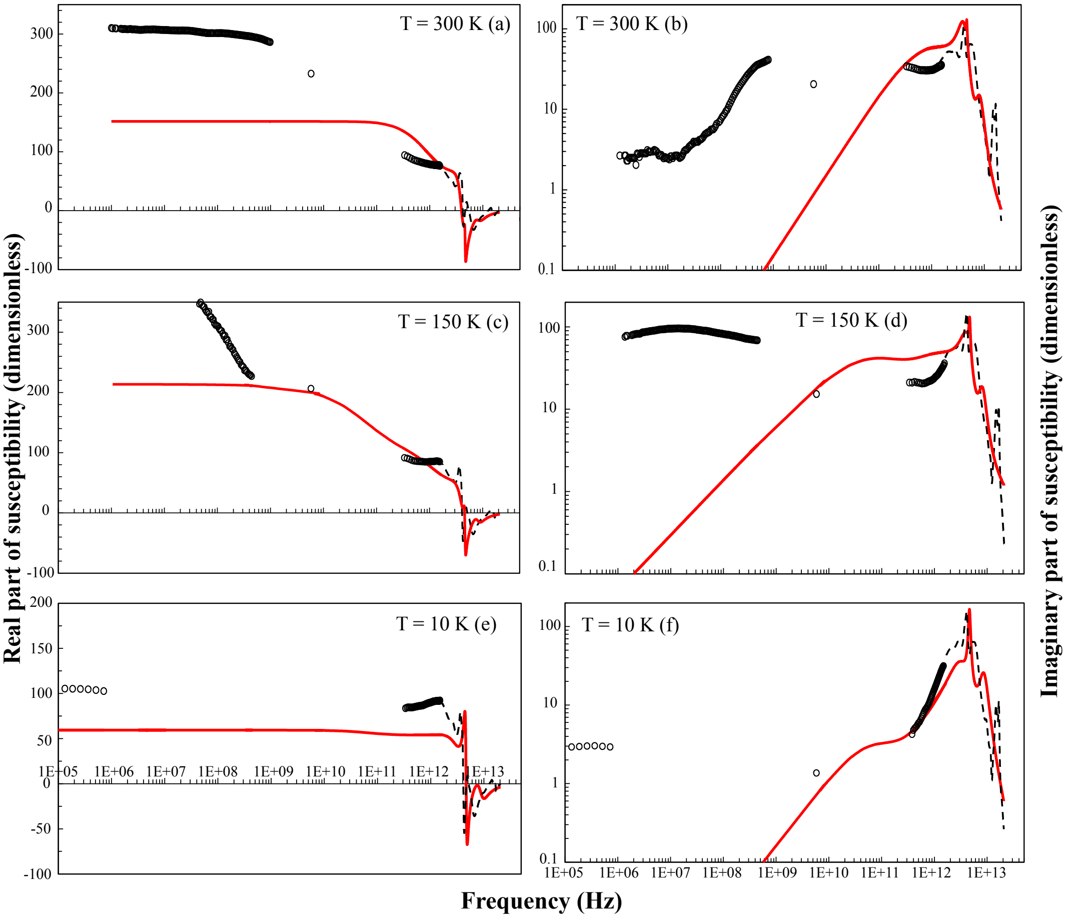

Supplementary Figure 3. Comparison of dielectric spectra. Here we compare the dielectric spectra obtained by MD simulations in $Ba(Ti_{0.5}Zr_{0.5})O_3$ and experimentally measured in $Ba(Ti_{0.4}Zr_{0.6})O_3$ ceramics. The black symbols are the data measured by a variety of dielectric spectroscopy techniques, the dashed black lines are the data obtained from IR reflectivity spectroscopy [5]. The red solid lines are the dependency calculated with Eq. (1) of the main text using the best-fit parameters.

Supplementary Note 1: Temperature evolution of dielectric spectra

This Note reports the parameters obtained by fitting the results of molecular dynamics (MD) simulations to Eq. (1) shown in the main text at different temperatures. For a better understanding of the contents, we remind the reader that $1\,\text{THz} \simeq 33\,\text{cm}^{-1}$, and the relation between the two units is strictly linear.

Supplementary Figure 1(a) shows the $1-\alpha$ and $(1-\alpha)\,\gamma$ coefficients of the Havriliak-Negami (HN) relaxation function, which determine the shape of relaxation spectrum at frequencies below and above ν_{HN}, respectively. Both of these coefficients are found to deviate from unity for temperatures below ~250 K, which indicates that this relaxation differs from a pure Debye mechanism. One can also see that these two parameters exhibit some fluctuations with temperature, which are most probably due to the fact that the fitting model described by Eq. (1) of the manuscript includes various overlapping contributions to the total dielectric response (note that the overlapping of these various contributions is also responsible for the fluctuations of other parameters shown in Supplementary Figure 1). It is also important to realize that Supplementary Figure 1(a) demonstrates that the $(1-\alpha)\,\gamma$ product is smaller than 1 for any temperature, as required for any meaningful description of the Havriliak-Negami relaxation [1, 2].

Supplementary Figure 1(b) further displays the oscillator strengths of the damped harmonic oscillators (DHOs), S_1 and S_2, and of the coupled oscillator-relaxator (COR), S_{CO}. S_1 and S_2 are rather insensitive to temperature, which contrasts with the case of S_{CO} that exhibits a broad peak around 200-400 K.

Moreover, Supplementary Figure 1(c) reports the damping constants of these DHOs and COR, as well as the coupling constant, δ, inherent to the COR mechanism. We have found that, for $T < 70$ K, the use of a heavily damped harmonic oscillator term instead of the COR term in Eq. (1) of the manuscript leads to better fitting. The change from COR to DHO model results in jumps in the temperature dependences of the related parameters at 70 K as observed in Supplementary Figure 1 (note that the DHO model is equivalent to the COR model with $\delta = 0$).

Furthermore, Supplementary Figure 1(d) shows the resonance frequencies of these DHOs and COR. Similar temperature behaviors were previously reported in Ref. [3] for the resonant frequencies of the Zr- and Ti-related DHOs (ν_1, ν_2, respectively), while the present addition of the COR mechanism (via its ν_{CO} and ν_{CR} frequencies) is found necessary here to further improve the fittings of the MD data.

Finally, Supplementary Figure 1(e) shows separate contributions of the HN relaxation, χ_{R0}, and of the COR mechanism, χ_{COR} to the total static susceptibility. It indicates that χ_{R0} vanishes above ~350 K, and narrowly peaks around 100 K. On the other hand, the temperature dependent peak of χ_{COR} is rather broad, centering around ~300 K and χ_{CO} is still significant for temperatures as high as 1000K. Note, however, and as cautioned in the manuscript, that the HN and COR contributions significantly overlap, especially at comparatively high temperatures. Consequently, some of their parameters can only be estimated rather than uniquely determined, which is also consistent with the fluctuations of $(1-\alpha)\,\gamma$ and ν_{CR} in Supplementary Figure 1.

Supplementary Note 2: Vogel-Fulcher law for the imaginary part of susceptibility

Supplementary Figure 2 shows the temperature of the maximum susceptibility, T_{m}, for the imaginary part. Here, we have numerically found that, for the imaginary part, the T_{m} versus ν curve can be fitted with the Vogel-Fulcher law, $\nu = \nu_0 \exp\left[-U/\left(T_{\mathrm{m}} - T_{\mathrm{VF}}\right)\right]$ (similar to what is done for the real part), but with a *negative* T_{VF} (the fitting parameters are: $\nu_0 = 3 \times 10^6\,\mathrm{cm}^{-1}$, $U = 6 \times 10^3$ K, and $T_{\mathrm{VF}} = -269$ K). Interestingly, this result fully agrees with Tagantsev's phenomenological theory [4] which predicts that the T_{VF} parameter of the imaginary part is smaller than that of the real part. Note also that T_{VF} can be negative in this theory as it is not a temperature that has physical significance; instead, it is simply a parameter that is determined by the temperature dependence of the dielectric strength and the parameters of the relaxation spectrum.

Let us now discuss in more detail the relation of our results to the published experimental data on $Ba(Zr_xTi_{1-x})O_3$ system. While no high-frequency measurements of the samples of exactly the same composition ($x = 0.5$) we theoretically investigate have been reported, the comparison with the available data of similar compositions ($x = 0.4$ and $x = 0.6$) is meaningful, as it is known that the characteristic relaxation frequencies and their temperature behaviour are similar for all the compositions with $0.2 \leq x \leq 0.8$ (see Figure 2 of Ref. [7] which shows the experimental results obtained for BZT ceramics). Significant differences only concern the magnitude and temperature variation of the static susceptibility [5, 6]. In Supplementary Figure 3, the available experimental spectra of BZT ceramics with $x = 0.4$ are compared to our MD predictions.

Similar to the MD results, two relaxation-related excitations were revealed in the THz and sub-THz ranges experimentally. The first one is an excitation with the characteristic frequency of ~ 3 THz (~ 100 cm^{-1}) which is practically temperature-independent (upper curve in Figure 2 of

Ref.[7]). It was successfully simulated with the coupled oscillator-relaxator (COR) at $T > 70$ K and the overdamped harmonic oscillator at lower temperatures (their frequencies $\nu_{\rm CR}$ and $\nu_{\rm CO}$ are shown in Supplementary Figure 1(d)). Another relaxation with a frequency of ~100 GHz, which we found to dominate in the subterahertz range for $T < T^*$, has also been observed experimentally. The characteristic frequency of this relaxation has not been determined accurately because of insufficiency of available experimental data points, but it has been found to remain within the range of 10 – 100 GHz at all studied temperatures (see Figure 2 of Ref. [7]). This experimental result is in agreement with our prediction that reveals the Arrhenius law at $T > 70$ K and the saturation of $\nu_{\rm m}$ at lower temperatures (see Figure 3 (c) in the main text). The predicted dielectric strength (contribution to the static susceptibility) of this mode, $\chi_{\rm R0}$, passes through a maximum upon cooling and practically vanishes at temperatures close to zero (see Supplementary Figure 1(e)). Similar behaviour has also been observed in experiments: as shown in Supplementary Figure 3(e), almost the same susceptibility is measured at 1 THz and at 1 MHz (i.e., well below and well above the experimentally determined characteristic frequency, respectively), which means that the relaxation strength at 10 K is much smaller than the relaxation strength at the temperature for which the real part of the static dielectric response is maximum.

Supplementary Note 3: Detailed comparison of MD simulation results with experiment

As shown in Supplementary Figure 3, in the THz and subterahertz parts of the spectrum, the results obtained from MD simulations agree well with experiments, not only for the characteristic frequencies, but also for the magnitude of the susceptibility. The most significant discrepancy is found for the magnitude at 10 K (85 and 60 from experiments and simulations, respectively). However, at $\nu \sim 1$ GHz (close to the lowest possible frequency available via MD simulations), the situation is more complex. While at 150 K the agreement is fairly good (cf. Supplementary Figure 3 (c)), at 300 K (cf. Supplementary Figure 3 (a)) the measured susceptibility is twice as large as the simulated one. The large increase of the measured susceptibility observed below ~ 10 GHz, which is not seen in simulations, is evidently due to the third relaxation mode having a characteristic frequency of ~ 1 GHz at 200-300 K (cf. Figure 2 of Ref. [7]). Upon cooling this mode moves to lower frequencies and disappears from the frequency window available in our simulation. We conclude, therefore, that this relaxation mode, which dominates the BZT's spectrum for frequencies lower than 1 GHz according to experiments, cannot be reproduced in our

MD simulations. For this reason the experimental and theoretical values of susceptibility can differ at $T > 150$ K below ~ 10 GHz. Note that, in addition to computational limitations, the possible discrepancy between the MD and experimental results may also come from the influence of defects (vacancies, dislocations, grain boundaries etc.) inherent to ceramic materials. It was found, in particular, that in relaxors the relaxation parameters can be appreciably different in ceramics and crystals (where the concentration of defects is generally smaller) [8]. Unfortunately, the dielectric spectra of BZT crystals have not been studied experimentally so far.

---

[1] Jonscher, A. *Universal Relaxation Law*. (Chelsea Dielectrics Press, 1996).

[2] Bokov, A. A., & Ye, Z.-G. Dielectric Relaxation in Relaxor Ferroelectrics. *J. Adv. Dielectr.* **2,** 1241010 (2012).

[3] Wang, D., Hlinka, J., Bokov, A. A., Ye, Z.-G., Ondrejkovic, P., Petzelt, J. & Bellaiche, L. Fano resonance and dipolar relaxation in lead-free relaxors. *Nat. Commun.* **5,** 5100 (2014).

[4] Tagantsev, A. Vogel-Fulcher relationship for the dielectric permittivity of relaxor ferroelectrics. *Phys. Rev. Lett.* **72,** 1100-1103 (1994).

[5] Petzelt, J., Nuzhnyy, D., Savinov, M., Bovtun, V., Kempa, M., Ostapchuk, T., Hlinka, J., Canu, G. & Buscaglia, V. Broadband dielectric spectroscopy of $Ba(Zr,Ti)O_3$: dynamics of relaxors and diffuse ferroelectrics. *Ferroelectrics* **469**, 14-25 (2014).

[6] Nuzhnyy, D., Petzelt, J. & Savinov, M. Broadband dielectric response of $Ba(Zr,Ti)O_3$ ceramics: from incipient via relaxor and diffuse up to classical ferroelectric behavior. *Phys. Rev. B* **86**, 014106 (2012).

[7] Petzelt, J., et al Lattice dynamics and dielectric spectroscopy of BZT and NBT lead-free perovskite relaxors - comparison with lead-based relaxors. *Phase Transitions* **88**, 320-332 (2015).

[8] Bovtun, V. et al Broad-band dielectric response of $PbMg_{1/3}Nb_{2/3}O_3$ relaxor ferroelectrics: Single crystals, ceramics and thin films. *J. Eur. Ceram. Soc.* **26**, 2867-2875 (2006).
